# Primary dysmenorrhea and its associated factors among female high school students in Nekemte town, East Wallaga Zone, Western Oromia, Ethiopia: a cross-sectional study

**DOI:** 10.3389/frph.2024.1451551

**Published:** 2025-01-07

**Authors:** Bekan Gudata Gindaba, Tesfaye Abera Gudeta, Lemane Dereje Sebu, Ebisa Zerihun Gindaba, Misgana Tesgara Abdisa

**Affiliations:** ^1^ School of Nursing and Midwifery, Wallaga University, Nekemte, Ethiopia; ^2^ Department of Nursing, Oda Bultum University, Chiro, Ethiopia; ^3^ Department of Nursing, Mettu University, Mettu, Ethiopia

**Keywords:** primary dysmenorrhea, associated factors, Nekemte, Ethiopia, high school

## Abstract

**Background:**

Primary dysmenorrhea is a common gynecological problem characterized by recurrent, periodical, and *cramping pain in the lower abdomen* that occurs before or during menstruation, usually without pelvic disease. Its magnitude has not been well studied; some of the associated factors are inconclusive. Therefore, the goal of this study was to fill gaps on the magnitude, and associated factors of primary dysmenorrhea among female high school students in Nekemte town.

**Objectives:**

To assess the magnitude of primary dysmenorrhea, its associated factors among high school students in Nekemte town, East Wallaga, Western Oromia, Ethiopia, 2023.

**Methods:**

An institutional-based cross-sectional study was conducted among high school students in Nekemte town from June 05 to 19, 2023. The calculated total sample size was 534, and data were collected from four governmental high schools and one private high school that were selected by multistage stratified sampling. The collected data were entered into Epi Info version 3.1 and analyzed using SPSS version 25. Binary and multivariable logistic regressions were used to find associations between dependent and independent variables.

**Results:**

The magnitude of primary dysmenorrhea was 68.4% (95% CI = 64.3%, 72.0%). Anxiety (AOR = 2.41, 95% CI = 1.31, 4.43), family history of primary dysmenorrhea (AOR = 4.64, 95% CI = 2.74, 7.86), sexual intercourse (AOR = 0.34, 95% CI = 0.21, 0.55), drinking tea <4 cups per day (AOR = 0.38, 95% CI = 0.22, 0.60), and physical activity (AOR = 0.06, 95% CI = 0.03, 0.11) were factors associated with primary dysmenorrhea.

**Conclusion:**

The magnitude of primary dysmenorrhea was high among high school students in the study area. Sexual intercourse, physical activity, drinking tea, anxiety, and family history of primary dysmenorrhea were significantly associated with primary dysmenorrhea.

## Introduction

Primary dysmenorrhea (PD) is a pain during the menstrual cycle characterized by recurrent, periodical and *cramping pain in the lower abdomen* that occurs before or during menstruation, usually without pelvic disease (1, 2). This abdominal pain usually starts 1–2 days before the onset of menses or just after the menstrual flow, typically lasts for 8–72 days (2, 3), and may radiate to the back and thighs (4, 5). In addition, the onset of primary dysmenorrhea usually occurs in adolescents and begins within a few months or within 2 years of menarche (3, 5).

Although the pathophysiological mechanisms of PD are not well established (6, 7), several studies have revealed that it is a complex process that may depend on hormonal many factors (8–10). Among those factors, prostaglandin plays a major role in the pathophysiological mechanism of PD (4–6).

Prostaglandins' production leads to muscle and vein contraction, which causes uterine ischemia and the production of anaerobic metabolites. As a result, pain during menses occurs. These prostaglandins are synthesized via the *arachidonic acid* mediated by the cyclooxygenase (COX) pathway. Progesterone levels control the *arachidonic acid*, which is mediated by COX pathways and is responsible for the production of prostaglandins (4, 6). In addition, vasopressin and other proinflammatory factors contribute to the development of primary dysmenorrhea (5, 7).

In addition to hormonal changes that occur in the body, non-hormonal factors, such as increasing age, early age at menarche, anxiety, length, and severity of menstruation, social disruption, drinking tea, sexual intercourse, marital status, and parity, may also contribute to the pathomechanism of PD (9–15). Moreover, many researchers propose that social, living, and psychology have added to this patho-mechanism of PD (4, 5, 8, 10).

PD is the most prevalent problem among adolescent girls (13, 16–20), globally, previous findings have reported that the magnitude of PD ranges from 51.1% to 92.3% of reproductive-age women, with 2%–29% experiencing severe pain, and a higher percentage (70%–90%) of younger women (<24 years of age) are generally reported (6, 21, 22). In Africa, the prevalence of PD varies from 51.1% to 78.35% (23, 24). In Ethiopia, the magnitude of PD ranges from 51.5% to 85.4% (10, 20).

Moreover, diagnose of PD does not require specializing in women's health or pelvic pain, and management can be started based on a typical history of painful menses without physical examination (3, 5).

In addition, different studies revealed that PD is the most frequently reported gynecological and menstrual problem and is the leading menstrual problem affecting 90% of adolescent girls and more than 50% of menstruating women (8, 10, 12, 25).

Additionally, primary dysmenorrhea is severe enough to result in significant socioeconomic problems, particularly in adolescents and young women (15). Different studies have shown that PD usually affects individuals' relationships, functioning, and productivity; contributes to absenteeism in class/work; and reduces daily life activities (11, 12, 26, 27). For example, in the United States, 600 million work hours and 2 billion dollars of cost-effective loss are likely associated with painful menses (3), and other studies have revealed that these losses have a significant negative impact on students' academic performance (10, 16, 27). Moreover, it also affects psychology, quality of life, and patterns of sleep and leads to central nervous system sensitization, which results in chronic pain syndrome (5).

In Ethiopia, a studies found that there are cultural taboos about menstruation like believing PD is a natural phenomenon, tolerated, and embarrassing (19, 28). In addition, the Ethiopia had School Health Program Framework which includes social and behavioral change communication and life skills development, school nutrition services, water, sanitation and hygiene (WASH) provision, Management of common infections, infestations, and disorders, Sexual and reproductive health services, and others but it did not incorporate about PD under sexual and reproductive health services (29).

Moreover, pain during menses is the most prevalent but conceivably the least recognized, neglected and least managed of all menstrual illnesses, and study participants may experience symptoms as a predictable response to menstruation (3, 4). So this study can help students, school authorities, and organizations working on school menstrual hygiene management program develop a better understanding of primary dysmenorrhea. In Ethiopia, specifically in Nekemte town among high school students, the magnitude of dysmenorrhea, especially primary dysmenorrhea, which is the most common problem, has not been well studied (10). Moreover, some of the factors associated with PD are inconclusive and controversial (11, 30). Therefore, the goal of this study was to assess the magnitude, and risk factors for primary dysmenorrhea among female high school students in Nekemte town.

## Methods and materials

### Study design, area, and period

Institution based cross sectional study was conducted in Nekemte town, East Wallaga Zone, Oromia Regional State, Ethiopia, from June 05 to 19, 2023. Nekemte is the capital city of the East Wallaga Zone and is located in the western part of the country at a distance of 324 km from Addis Ababa, the capital city of Ethiopia. According to the report of the Nekemte town educational bureau, there were 16,379 high school students, of which 8,887 were female students and 7,492 were male students. In the town, there are eight public high schools namely, Nekemte secondary school, Biftu Nekemte secondary school, Dalo secondary school, Darge secondary school, Dire Jato secondary school, Kumsa Moroda secondary school, Leka Nekemte secondary school, and Ifa Boru Boarding School. In addition, there are three private high schools in the town, namely, Bethel Academy, Kidanemihiret (Catholic Academy), and Onesmosnasib Academy.^1^

### Population, sample size, and sampling technique

All female high school students in Nekemte town who were pursuing their education were the source population. On other hand, all female students from five selected high schools in Nekemte town were the study population. The inclusion criterion for this study was all female students from five selected high schools. However, a female student who had a known pelvic pathology was excluded from thew study which was reported by the students.

The sample size was determined using Epi Info 7 by taking the expected proportion of primary dysmenorrhea (70%) from a study of Wolaita Sodo Town Secondary School Students (31) with a 5% margin of error and a 95% confidence interval. Considering the design effect of 1.5 and a nonresponse rate of 10%, the total sample size was 534. Study participants were selected from all Nekemte town high schools using a multistage stratified sampling technique. First, the eleven schools were stratified into eight public and three private schools. Then, the public schools were classified based on their location as East (Nekemte Secondary Shool and Dalo Secondary School), West (Leka Nekemte Secondary School and Ifa Boru Boarding School), South (Biftu Nekemte Secondary School, Dire Jato Secondary School, and Darge Secondary School), and North (Kumsa Moroda Secondary School). Again from all directions one school was selected by lottery method. The selected four Schools were Nekemte Secondary School; Biftu Nekemte Secondary School; Leka Nekemte Secondary School; and Kumsa Moroda Secondary School. Finally, we selected one from a private school, Bethel Academy. The selected schools were again stratified and a new sampling frame were constructed. Finally, the study participants were selected using a simple random sampling technique via the lottery method.

### Study variables

The dependent variable was primary dysmenorrhea. Whereas, **socio-demographic factors** included age, marital status, residence, educational status of the mother, and educational status of the father, **psychosocial and behavioral factors:** smoking, physical exercise, attempted weight loss, anxiety, drinking coffee, drinking tea, drinking soft drinks, chocolate consumption, sexual intercourse, and social disruption, **reproductive factors** included age at menarche, parity, menses flow, menses interval, duration of menses, and family history of dysmenorrhea were independent variables.

### Operational definition

**Primary dysmenorrhea** was considered to indicate PD if she said “Yes” about pain during menses and had one or more of the following complaints: abdominal pain, groin pain, pelvic pain, back pain, or thigh pain before and/or during her menstrual periods within the past 6 months (15).

**The visual analog scale (VAS)** was used to assess students' degree of menstrual pain on a 10-cm line. One extremity of the line represented “unimaginably pain”, and the other extremity represented “no pain at all”. The participants were asked to rate the degree of pain by making a mark on the line. Study participants were classified as mild pain if they were between 1 and 3 points, moderate if they were between 4 and 7 points, and severe if they were between 8 and 10 points (32).

**Physical activity**: The habit of physical activity was measured by the participants' self-reports of not at all, irregularly or regularly (31).

**The menstrual characteristics** were as follows: long cycle: cycle returning once every >35 days; short (frequent) cycle: cycle recurring once every <21 days; short duration: duration of menses <3 days; and long duration: duration of period >7 days. Heavy menses were considered if a student changed 3 or more sanitary/vulvar pads per day but was scant if one or less (10, 15).

**Social disruption** was considered if a student said yes to trouble in social networks such as with family, friends, or people you loved in the past 6 months.

**Sexually active**: considered if students had at least one genital-to-genital contact.

**Family history:** Study participants with a positive family history of PD, where a first-degree relative (either mother, sister or grandmother) had a history of menstrual pain (33).

Attempted Weight loss: The habit of attempted weight loss was measured by the participants' self-reports of yes or no.

### Ethical concern

Ethical approval was obtained from the Institutional Review Board (IRB) of Wallaga University (WU). The selected school's directors were formally requested with a written letter to ensure their consent before starting the data collection. Written and signed consent and assent was obtained from the students regarding their agreement to participate in the study after the objective of the study was explained to them. All appropriate ethical principles under the Helsinki declaration were followed and respected.

### Data quality assurance and analysis

The quality of the data was ensured during collection, coding, entry, and analysis. The principal investigator and supervisor were included in the observation of how the study participants administered the questionnaires. The questionnaire and each School's section during the data collection were coded so that any identified errors could be traced back using the codes.

The facilitators, supervisors, and principal investigators checked the questionnaire daily for completeness. The pretest was administered to 27 (5%) female Gute High School students. In addition, experts in the field checked the faces' validity.

The questionnaire was first manually checked for completeness, after which the data were coded, entered in Epi Data version 3.1 and subsequently exported to SPSS version 25 for data analysis. Descriptive statistics, such as frequency, percentage, mean, and standard deviation, were used to describe the study population and their management practices. Both bivariable and multivariable logistic regression analyses were used to identify factors associated with the outcome variable. For the bivariable logistic regression, the crude odds ratio (COR) and 95% CI were calculated, and for the multivariable logistic regression, the adjusted odds ratio (AOR) and 95% CI were calculated. In the bivariable analysis, all variables with *p* values less than 0.25 were considered candidates for multivariate analysis (19). Hosmer and Lemeshow's goodness-of-fit test was performed to assess whether the required assumptions were fulfilled, and the variance inflation factors were used to assess the presence of multicollinearity. An adjusted odds ratio (OR) with 95% confidence intervals (CIs) and a *p* value less than 0.05 were considered to indicate a statistically significant association. Finally, text, tables, and graphs were used to present the results.

## Results

### Sociodemographic characteristics

In this study, 534 self-administered questionnaires were distributed, and almost all of the questionnaires were completed, for a response rate of 532 (99.6%). Two participants did not voluntarily participate in the study. The students involved in the study were aged between 15 and 22 years, with a mean age and standard deviation of 17.76 ± 1.5 years.

With regard to the ethnicity of the study participants, five hundred (94.0%) were Oromo, and 370 (69.5%) were protestant in religion. Five hundred fifteen (96.8%) and 480 (90.2%) of the study participants were single and urban residents, respectively.

Data relating to parents' educational status showed that 144 (27.1%) of the fathers had a diploma and above, while only 73 (13.7%) of the mothers had a diploma and above (Table 1).

**Table 1:** Sociodemographic characteristics of female high school students in Nekemte Town, Western Oromia, Ethiopia, 2023.

Variables	Category	Frequency	Percentage
Age	15–17	248	46.6%
	18–19	218	41.0%
	20–22	66	12.4%
	Total	532	100%
Grade	Grade 9th	153	28.8%
	Grade 10th	124	23.3%
	Grade 11th	114	21.4%
	Grade 12th	141	26.5%
	Total	532	100%
Marital status	Single	515	96.8%
	Married	17	3.2%
	Total	532	100%
Ethnicity	Oromo	500	94.0%
	Amhara	17	3.2%
	Others^a^	15	2.8%
	Total	532	100%
Religion	Protestant	370	69.5%
	Orthodox	100	18.8%
	Muslim	46	8.6%
	Others^b^	16	3.0%
	Total	532	100%
Residence	Urban	480	90.2%
	Rural	52	9.8%
	Total	532	100%
Educational background of father	I don't know	21	3.9%
	Unable to read and write	69	13.0%
	Read and write	49	9.2%
	Primary school	121	22.7%
	Secondary school	128	24.1%
	Diploma and above	144	27.1%
	Total	532	100%
Educational background mother	I don't know	22	4.1%
	Unable to read and write	74	13.9%
	Read and write	119	22.4%
	Primary school	161	30.3%
	Secondary school	83	15.6%
	Diploma and above	73	13.7%
	Total	532	100%

^a^ Others—Walaita, Gurage, Tigre, and Silte.

^b^ Others—Catholic, Adventist, and Wakefata.

### Psychosocial and behavioral characteristics

Among the study participants, 141 (26.5%) attempted weight loss, and 231 (43.4%) started sexual intercourse. Concerning physical activity, approximately two-tenths of the 105 (19.7%) participants exercised regularly, and none of them chewed khat or smoked cigarettes.

Regarding drinking habits, more than nine-tenths (484; 91.0%) of the study participants had never drank alcohol, approximately one-third (164; 30.8%) had never drank tea at all, and nearly half (260; 48.8%) had never drank coffee at all.

More than half (278; 52.3%) of the study participants did not drink soft drinks, and 246 (46.2%) of them did not consume chocolate.

Regarding the history of anxiety of the study participants, the majority (443; 83.3%) had a history of anxiety, and more than half (236; 55.6%) of them did not have a history of social disruption, such as family, friends or loved ones, in the past 6 months (Table 2).

**Table 2:** Psychosocial and behavioral characteristics of female high school students in Nekemte Town, Western Oromia, Ethiopia, 2023.

Variables	Categories	Frequency	Percentage
History of attempt to weight loss	Yes	141	26.5%
	No	391	73.5%
	Total	532	100%
Sexually active	Yes	231	43.4%
	No	301	56.6%
	Total	532	100%
Have sexual intercourse in the past 6 month	Yes	148	64.1%
	No	83	35.9%
	Total	231	100%
Physical activity	No	279	52.4%
	Regularly	105	19.7%
	Irregularly	148	27.8%
	Total	532	100%
Smoking cigarette	Yes	0	0
	No	532	100%
Drinking alcohol	No	484	91.0%
	Yes, I drunk occasionally(2–3) times in a month	26	4.9%
	Yes, I drunk daily	22	4.1%
	Total	532	100%
Chewed khat	Yes	0	0
	No	532	100%
Drinking tea	No	164	30.8%
	Yes, <four glass per day	306	57.5%
	Yes, ≥four glass per day	62	11.7%
	Total	532	100%
Drinking coffee	No	260	48.9%
	Yes, I drunk <three cup per day	245	46.1%
	Yes I drunk ≥three cup per day	27	5.1%
	Total	532	100%
History of anxiety	Yes	443	83.3%
	No	89	16.7%
	Total	532	100%
Disruption of social networks like with family, lover etc.	Yes	236	44.4%
	No	296	55.6%
	Total	532	100%
Drinking soft drinks	No	278	52.3%
	Yes, I drunk one soft drinks per day	222	41.7%
	Yes, I drunk more than one soft drinks per day	32	6.0%
	Total	532	100%
Chocolate consumption	No	246	46.2%
	<2 bars of chocolate per day	242	45.5%
	≥2 bars of chocolate per day	44	8.3%
	Total	532	100%

### Reproductive characteristics

The mean age at menarche of the study participants was 13.11 ± 1.18 years (SD). Among them, more than half (317; 59.6%) started their first menstruation between the ages of 13 and 14 years.

Two hundred seventy-one (50.9%) of the study participants had irregular menstruation, and more than half (304; 57.1%) of the menstrual cycle was between 21 and 35 days, which lasted for 2–5 days in 375 (70.5%) of the study participants. Three hundred and sixty-six (68.8%) of them had changed ≥3 pads per day per cycle.

Concerning the family history of the study participants, 226 (42.5%) had a family history of PD, and 509 (95.7%) had never given birth (Table 3).

**Table 3:** Reproductive characteristics of female high school students in Nekemte Town, Western Oromia, Ethiopia, 2023.

Variables	Categories	Frequency	Percentage
Age at menarche	≤12	154	28.9%
	13–14	317	59.6%
	≥15	61	11.5%
	Total	532	100%
Duration of Menstruation	<2 day	67	12.6%
	2–5 days	375	70.5%
	>5 days	90	16.9%
	Total	532	100%
Interval of menstruation	<21 days	106	19.9%
	21–35 days	371	69.7%
	>35 days	55	10.3%
	Total	532	100%
Regularity of menstruation	Regular	261	49.1%
	Irregular	271	50.9%
	Total	532	100%
Number of pad changed per day per cycles	<3 pads/day	166	31.2%
	≥3 pads/day	366	68.8%
	Total	532	100%
Parity	Multipara	23	4.3%
	Nullipara	509	95.7%
	Total	532	100%
Family history of dysmenorrhea	I do not know	11	2.1%
	Yes	226	42.5%
	No	295	55.5%
	Total	532	100%
Positive family history	Mother	123	54.4%
	Sister	94	41.6%
	Grand mother	9	4.0%
	Total	226	100.0%

### The magnitude and menstrual characteristics of PD

**Figure 1:**
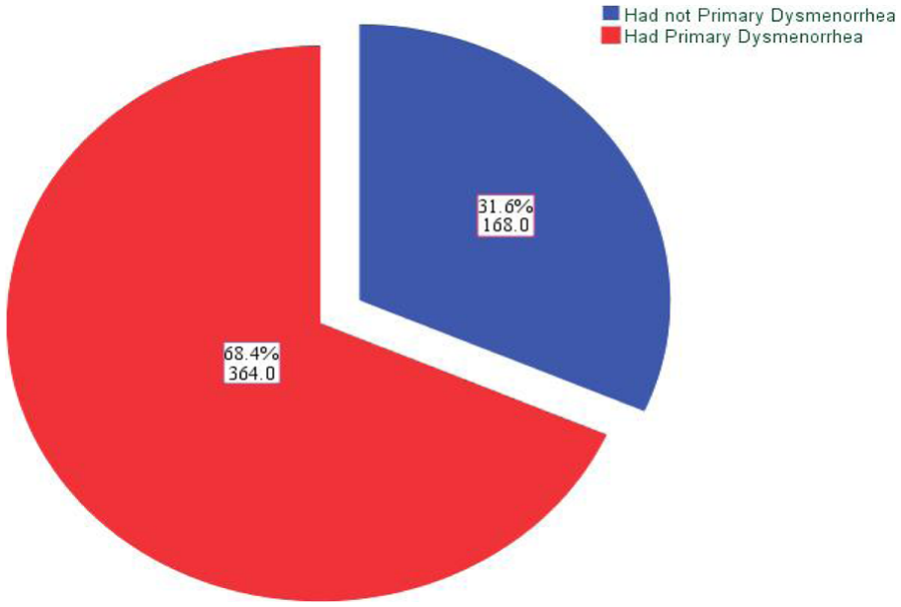
Magnitude of primary dysmenorrhea among female high school students in Nekemte Town, East Wallaga, Western Oromia, Ethiopia, 2023.

In this study, the magnitude of PD was 364 (68.4%) (95% CI (64.3%, 72.0%). Among the students who had PD, the majority (249; 68.4%) had moderate to severe pain (Figure 1).

Among the dysmenorrheic study participants, 96 (26.4%) had been missing from school in the past 6 months due to pain during menstruation.

**Figure 2:**
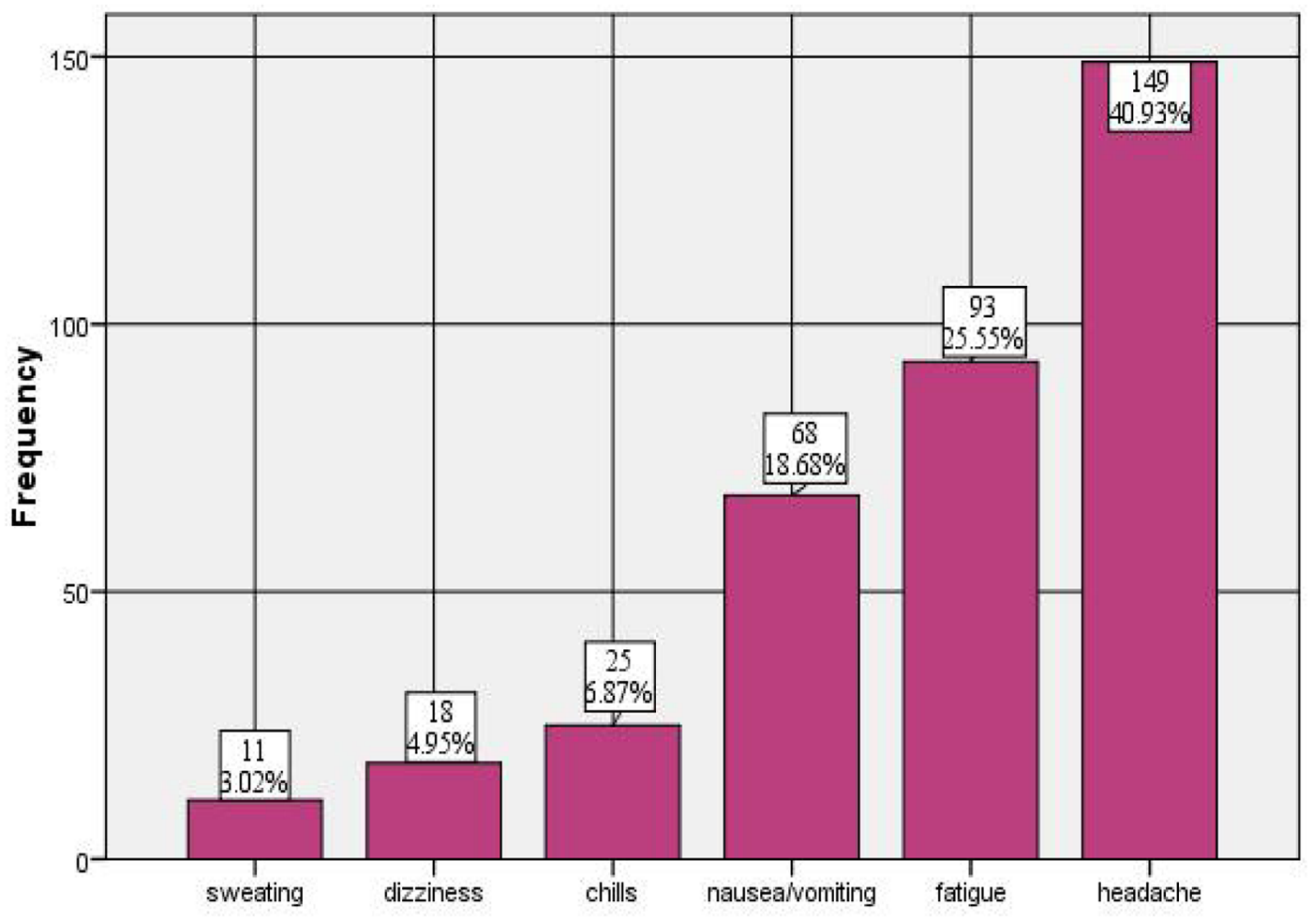
Symptoms of primary dysmenorrhea among female high school students in Nekemte Town, Western Oromia, Ethiopia, 2023.

Of the dysmenorrhic study participants, 157 (43.1%) had started experiencing pain within 3 months of puberty; 159 (43.7%) had pain starting from the first day of menses, which persisted for 2–3 days 288 (79.1%) and was located in the lower back 232 (63.7%). In addition, 149 (40.9%) of the study participants experienced headache, and 117 (32.1%) felt restless or anxious during their painful period (Table 4), (Figure 2).

**Table 4:** Magnitude and menstrual characteristics of female high school students in Nekemte Town, Western Oromia, Ethiopia, 2023.

Variables	Categories	Frequency	Percentage
Intensity of pain	Mild pain	115	31.6%
	Moderate pain	188	51.6%
	Severe pain	61	16.8%
	Total	364	100%
School absenteeism	Yes, 1 day	46	12.6%
	Yes, 2 day	37	10.2%
	Yes, 3–4 day	13	3.6%
	No	268	73.6%
	Total	364	100%
School absenteeism per cycle	1 day/cycle	78	81.2%
	2 day/cycle	15	15.6%
	3 day/cycle	3	3.1%
	Total	96	100%
When starts experiencing painful period	Within 3 months of puberty	157	17.9%
	Within 6 months of puberty	90	24.7%
	Within 9 months of puberty	12	3.3%
	Within 1 year of puberty	39	10.7%
	Within 2 year of puberty	35	9.6%
	Within 3 years of puberty	31	8.5%
	Total	364	100%
When the pain starts	Three days before menses begins	65	17.9%
	Two days before menses begins	59	16.2%
	One day before menses begins	71	19.5%
	First day of menses	159	43.7%
	Second day of menses	7	1.9%
	Third day of menses	3	0.8%
	Total	364	100%
Duration of pain	<2 days	67	18.4%
	2–3 days	288	79.1%
	>3 days	9	2.5%
	Total	364	100%
Site of pain	Lower back	232	63.7%
	Abdominal	25	6.9%
	Inner thigh	90	24.7%
	Breast	17	4.7%
	Total	364	100%
Emotional symptoms	No change	83	22.8%
	Angry/irritable	97	26.6%
	Restless/anxious	117	32.1%
	Depressed/sad	42	11.5%
	Socially embarrassed	25	6.9%
	Total	364	100%

### Bivariate and multivariable logistic regression analysis

The results from the multivariate logistic regression analysis showed that sexual intercourse, drinking tea, history of anxiety, family history, and physical activity were variables that were significantly related to primary dysmenorrhea.

The results of the analysis showed that the odds of having PD were 4.64 times greater for students with a positive family history of primary dysmenorrhea than for those who did not have a family history of PD (AOR 4.64, 95% CI: 2.74, 7.86). Similarly, the odds of having PD were 2.41 times greater for those with a history of anxiety than for their counterparts (AOR 2.41, 95% CI = 1.31, 4.43).

Students who performed regular physical activities were 0.06 less likely to develop PD than were those who did not perform any physical activities (AOR 0.06, 95% CI = 0.03, 0.11). In addition, students who drank less than four glasses per day were 0.38 less likely to develop PD than were those who did not drink tea at all (AOR = 0.38, 95% CI = 0.22, 0.60). In addition, those who had sexual intercourse were 0**.**34% less likely to develop PD than were those who had not had sexual intercourse in the past 6 months (AOR = 0.34, 95% CI = 0.21, 0.55) (Table 5).

**Table 5:** Multiple logistic regression results for female high school students in Nekemte Town, Western Oromia, Ethiopia, 2023.

Variables	Category	PD		COR(95% CI)	AOR(95%CI)	*P* value
		Yes	No			
Age	15–17	178	70	1.99 (1.14, 3.49)	1.40 (0.65, 3.04)	0.392
	8–19	149	69	1.69 (0.96, 2.97)	1.10 (0.50, 2.42)	0.805
	20–22	37	29	**1**	**1**	
Sexual intercourse	Yes	131	100	0.38 (0.26, 0.56)	0.34 (0.21, 0.55)	**<0**.**001**^a^
	No	233	68	**1**	**1**	
Drinking tea	No	122	42	**1**	**1**	
	<Four glass per day	190	116	0.56 (0.37, 0.86)	0.38 (0.22, 0.66)	**0**.**001**^a^
	≥Four glass per day	52	10	1.79 (0.83, 3.84)	1.65 (0**.**66, 4.07)	0.281
Physical activities	No	222	57	**1**		**<0.001** ^a^
	Regularly	23	82	0.07 (0.04, 0.12)	0.06 (0.03, 0.11)	
	Irregularly	119	29	1.05 (0.64, 1.74)	1.13 (0.65, 1.95)	0.670
Social disruption like family, lover	Yes	170	66	1.35 (0.93, 1.96)	1.05 (0.63, 1.75)	0.848
	No	194	102	**1**	**1**	
History of anxiety	Yes	313	130	1.79 (1.12, 2.86)	2.41 (1.31, 4.43)	**0**.**004**^a^
	No	51	38	**1**	**1**	
Educational background of father	I don't know	11	10	0.45 (0.18, 1.15)	0.80 (0.23, 2.77)	0.722
	Unable to read and write	48	21	0.94 (0.50, 1.76)	1.67 (0.68, 4.13)	0.265
	Read and write	30	19	0.65 (0.33, 1.28)	0.86 (0.33, 2.23)	0.756
	Primary school	88	33	1.09 (0.64, 1.88)	2.05 (0.95, 4.45)	0.068
	Secondary school	85	43	0.81 (0.49, 1.36)	1.31 (0.64, 2.70)	0.455
	Diploma and above	102	42	**1**	**1**	
Educational background of mother	I don't know	13	9	0.28 (0.09, 0.81)	0.29 (0.07, 1.11)	0.071
	Unable to read and write	52	22	0.46 (0.21, 1.03)	0.53 (0.20, 1.45)	0.218
	Read and write	75	44	0.33 (0.16, 0.69)	0.38 (0.15, 1.19)	0.240
	Primary school	104	57	0.36 (0.18, 0.72)	0.45 (0.18, 1.09)	0.078
	Secondary school	59	24	0.48 (0.22, 1.05)	0.39 (0.15, 1.05)	0.064
	Diploma and above	61	12	**1**	**1**	
Family history of PD	I do not know	5	6	0.64 (0.19, 2.14)	1.01 (0.17, 5.86)	0.988
	Yes	192	34	4.33 (2.81, 6.66)	4.64 (2.74, 7.86)	**<0**.**001**^a^
	No	167	128	**1**	**1**	
History of attempt to weight loss	Yes	107	34	1.64 (0.06, 2.54)	1.56 (0.88, 2.75)	0.126
	No	257	134	**1**	**1**	
Age at menarche	<12	106	48	1.43 (0.77, 2.65)	1.49 (0.66, 3.40)	0.338
	13–14	221	96	1.49 (0.85, 2.63)	1.78 (0.82, 3.86)	0.145
	>15	37	24	**1**	**1**	
Marital status	Single	357	158	3.23 (1.23, 8.63)	2.47(0.60, 10.23)	0.213
	Married	7	10	**1**	**1**	

Key: **1**- Reference group.

^a^ Significantly associated.

## Discussion

Primary dysmenorrhea is often disregarded by affected adolescents who believe pain to be a normal part of the menstrual cycle. Thus, many study participants fail to seek treatment (6). It has adverse consequences for personal, family, and social life, resulting in short-term school and work absenteeism (3, 10). Having a better understanding of this problem will improve the understanding of primary dysmenorrhea among adolescents even at the family, school and community levels and help to improve the quality of life and academic performance of adolescent girls. Therefore, this study aimed to assess the magnitude of primary dysmenorrhea, its associated factors, and management practices among female high school students in Nekemte town, Western Oromia, Ethiopia.

This study showed that the magnitude of primary dysmenorrhea was 68.4%, which means that out of 100 students, 68 had PD. This magnitude was in line with previous studies conducted in the East Hararghe Zone (69.3%) (31), Wolaita Sodo town (70%) (19), Haramaya University (64.7%) (34), Debremarkos town (69.26%) (15) and Debra Berhan University (66.8%) (27) in Ethiopia and Ghana (68.1%) (35).

However, this percentage was slightly greater than that reported in studies conducted at Hawassa University (51.5%) (20) in Ethiopia; Enugu State; Nigeria (51.1%) (24); and Chinese college students (51.1%) (22). This variation may be due to differences in the operational definitions, study population, age and factors like diet, local health care and stress. This implies that PD affects a significant proportion of the students, so health policy may be applied in Schools.

On the other hand, the prevalence of bias in this population was slightly lower than that reported in studies of Gondar university students (77.6%) (14) and Debra Berhan University (85.4%) in North Ethiopia (10); Benin (78.35%) (23); Ibadan State; Nigeria (73%) (12); and Parakou, Benin (72.6%) (36). Furthermore, the proportions of studies conducted in Riyadh, King Dom of Saudi Arabia (92.3%) (21), King Saud University, Riyadh, Saudi Arabia (80.1%) (18), Iran (89.1%) (37), and Lebanon (80.9%) (33) were much greater than those in this study. A reason for the variation in this study could be age variation and the operational definition of primary dysmenorrhea; many studies use the definition of dysmenorrhea in general. Another reason for the variation could be differences in sociocultural, ethnic and lifestyle factors among the study participants.

In this study, the odds of PD were approximately 2.41 times greater for students who had a history of anxiety than for those with no history of anxiety. This finding was supported by studies performed at Haramaya University, Eastern Ethiopia, and Debra Berhan University, North Ethiopia (10, 13). However, no statistically significant association was reported between anxiety and PD in Hawassa University students (20). This may be because of differences in the measurements used; i.e., in this study, anxiety was measured by students' self-reports as yes or no for the past 6 months, whereas at Hawassa University, students were asked about anxiety only related to tests or assignments. Therefore, it would be helpful if the schools taught students ways of overcoming stress, like deep relaxation, muscle relaxation (exercise), cultivating positive relationships with their friends and teachers, and engaging in enjoyable activities.

This study revealed that sexual intercourse decreased the risk of primary dysmenorrhea by 66% compared with that of their counterpart, which is comparable with the findings of studies performed in Debremarkos town, northwestern Ethiopia (15). The reason may be that during the orgasm of sexual intercourse, the muscle of the uterus contracts and then relaxes, which leads to some relief from pain. In addition, sex activates the release of chemical endorphins, which make individuals feel good, and engaging in sexual activity exacerbates menstrual discomfort (38).

In this study, students with a family history of dysmenorrhea had an approximately 4.6-fold greater risk of developing PD than did those without dysmenorrhea. These findings are in line with those of other studies from Ethiopia (10, 15, 19, 20, 31), Iran (37), Lebanon (33), Saudi Arabia (16), and Australia (30). This could be related to coping behaviors from mothers to control pain. A genetics might also have a psychological impact, as girls may react to menstruation similarly to their mothers, and they may share the same outlook on menstruation (39). Therefore, there would be a strategy for educating families of students about PD.

This study revealed that students who drank tea tended to experience primary dysmenorrhea alleviation. Those who consumed <4 cups/day of tea were 53% less likely to report having mild PD than nondrinkers were. Different studies support these results; case control study done at Debra Berhan town (40), cross sectional study Debra Berhan university (10), and Shanghai, China (41). However, other studies contradict this finding: a study was performed at Debre Markos town (15) and Haramaya University (13). This possibility requires further study.

In this study, regular physical activity was found to be significantly associated with the occurrence of PD (AOR = 0.06; 95% CI = 0.22, 0.60), which was in line with the findings in Debre Markos town (15) and Lebanon (33). Nevertheless, no statistically significant associations were reported between physical activity and PD in Saudi Arabia (18) or at Haramaya University (13). This difference may be because of differences in physical activity. In this study, physical activity was self-reported by the participants as not at all, irregularly, or regularly. Similarly, in Saudi Arabia, participants were asked whether they had not participated, 1–2 times per week, 3–4 times per week, or 5–6 times per week and daily, respectively. In addition, Haramaya University asked respondents if they were yes or no questions. This reqquires further studies.

### Limitation of the study

The diagnosis of primary dysmenenorrhea was made only by history of the students.

Recall bias and over or under reporting of the condition may be present.

## Conclusion

In this study, the overall magnitude of primary dysmenorrhea among Nekemte town high school students was found to be high.

Individuals with a positive family history and anxiety were more likely to be symptomatic, and those who drank <4 cups of tea per day and who had sexual intercourse and physical activity were less likely to have primary dysmenorrhea.

## Data Availability

The original contributions presented in the study are included in the article/Supplementary Material, further inquiries can be directed to the corresponding author.

## Footnotes

^1^Bureau NE. Data of Nekemte town high school students (2023).

## References

[1] Burnett M Lemyre M. No. 345-Primary dysmenorrhea consensus guideline. J Obstet Gynaecol Can. (2017) 39(7):585–95. 10.1016/j.jogc.2016.12.023 28625286

[2] Committee on Adolescent Health Care, Geri D Hewitt MA Karen R Gerancher M. ACOG committee opinion no. 760: dysmenorrhea and endometriosis in the adolescent. Obstet Gynecol. (2018) 132(6):e249–58. 10.1097/AOG.0000000000002978 30461694

[3] Kho KA Shields JK. Diagnosis and management of primary dysmenorrhea. JAMA. (2020) 323(3):268–9. 10.1001/jama.2019.16921 31855238

[4] Iacovides S Avidon I Baker FC. What we know about primary dysmenorrhea today: a critical review. Hum Reprod Update. (2015) 21(6):762–78. 10.1093/humupd/dmv039 26346058

[5] Ferries-Rowe E Corey E Archer JS. Primary dysmenorrhea. Obstet Gynecol. (2020) 136(5):1047–58. 10.1097/AOG.0000000000004096 33030880

[6] Itani R Soubra L Karout S Rahme D Karout L Khojah HMJ. Primary dysmenorrhea: pathophysiology, diagnosis, and treatment updates. Korean J Fam Med. (2022) 43(2):101–8. 10.4082/kjfm.21.0103 35320895 PMC8943241

[7] Barcikowska Z Rajkowska-Labon E Grzybowska ME Hansdorfer-Korzon R Zorena K. Inflammatory markers in dysmenorrhea and therapeutic options. Int J Environ Res Public Health. (2020) 17(4):1191. 10.3390/ijerph17041191 32069859 PMC7068519

[8] Bernardi M Lazzeri L Perelli F Reis FM Petraglia F. Dysmenorrhea and related disorders. F1000Res. (2017) 6:1645. 10.12688/f1000research.11682.1 28944048 PMC5585876

[9] Sharghi M Mansurkhani SM Larky DA Kooti W Niksefat M Firoozbakht M An update and systematic review on the treatment of primary dysmenorrhea. JBRA Assist Reprod. (2019) 23(1):51–7. 10.5935/1518-0557.20180083 30521155 PMC6364281

[10] Hailemeskel S Demissie A Assefa N. Primary dysmenorrhea magnitude, associated risk factors, and its effect on academic performance: evidence from female university students in Ethiopia. Int J Women’s Health. (2016) 8:489–96. 10.2147/IJWH.S112768 27695366 PMC5034908

[11] Al-Matouq S Al-Mutairi H Al-Mutairi O Abdulaziz F Al-Basri D Al-Enzi M Dysmenorrhea among high-school students and its associated factors in Kuwait. BMC Pediatr. (2019) 19(1):80. 10.1186/s12887-019-1442-6 30885151 PMC6421654

[12] Femi-Agboola DM Sekoni OO Goodman OO. Dysmenorrhea and its effects on school absenteeism and school activities among adolescents in selected secondary schools in Ibadan, Nigeria. Niger Med J. (2017) 58(4):143–8. 10.4103/nmj.NMJ_47_17 31057207 PMC6496977

[13] Mesele TT Dheresa M Oljira L Wakwoya EB Gemeda GM. Prevalence of dysmenorrhea and associated factors among haramaya university students, eastern Ethiopia. Int J Women’s Health. (2022) 14:517–27. 10.2147/IJWH.S333447 35440875 PMC9013413

[14] Gebeyehu MB Mekuria AB Tefera YG Andarge DA Debay YB Bejiga GS Prevalence, impact, and management practice of dysmenorrhea among university of gondar students, northwestern Ethiopia: a cross-sectional study. Int J Reprod Med. (2017) 2017:1. 10.1155/2017/3208276 PMC5446888 28589173

[15] Muluneh AA Nigussie TS Gebreslasie KZ Anteneh KT Kassa ZY. Prevalence and associated factors of dysmenorrhea among secondary and preparatory school students in debremarkos town, north-west Ethiopia. BMC Women’s Health. (2018) 18(1):57. 10.1186/s12905-018-0552-x 29699536 PMC5921558

[16] Ali A Ali A Alotaibi N Alsufyani M Alotaibi A Almutairi M Prevalence, impact, and management perception of dysmenorrhea among university students: a cross-sectional study. Braz J Pharm Sci. (2022) 58. 10.1590/s2175-97902022e20458

[17] de Las Mercedes Villa Rosero CY Mazin SC Nogueira AA Vargas-Costales JA Rosa ESJC Candido-Dos-Reis FJ Prevalence of chronic pelvic pain and primary dysmenorrhea in women of reproductive age in Ecuador. BMC Womens Health. (2022) 22(1):363. 10.1186/s12905-022-01948-y 36056424 PMC9438184

[18] Hashim RT Alkhalifah SS Alsalman AA Alfaris DM Alhussaini MA Qasim RS Prevalence of primary dysmenorrhea and its effect on the quality of life amongst female medical students at king saud university, Riyadh, Saudi Arabia. A cross-sectional study. Saudi Med J. (2020) 41(3):283–9. 10.15537/smj.2020.3.24988 32114601 PMC7841556

[19] Mammo M Alemayehu M Ambaw G. Prevalence of primary dysmenorrhea, its intensity and associated factors among female students at high schools of Wolaita Zone, Southern Ethiopia: cross-sectional study design. Int J Women’s Health. (2022) 14:1569–77. 10.2147/IJWH.S384275 36387327 PMC9656336

[20] Tadese M Kassa A Muluneh AA Altaye G. Prevalence of dysmenorrhoea, associated risk factors and its relationship with academic performance among graduating female university students in Ethiopia: a cross-sectional study. BMJ Open. (2021) 11(3):e043814. 10.1136/bmjopen-2020-043814 33741669 PMC7986900

[21] Bakhsh H Algenaimi E Aldhuwayhi R AboWadaan M. Prevalence of dysmenorrhea among reproductive age group in Saudi women. BMC Women’s Health. (2022) 22(1):78. 10.1186/s12905-022-01654-9 35305636 PMC8933932

[22] Chen L Tang L Guo S Kaminga AC Xu H. Primary dysmenorrhea and self-care strategies among Chinese college girls: a cross-sectional study. BMJ Open. (2019) 9(9):e026813. 10.1136/bmjopen-2018-026813 31537555 PMC6756436

[23] Sidi I Hounkpatin B Obossou AAA Salifou K Vodouhe M Denakpo J Primary dysmenorrhea in the schools of Parakou: prevalence, impact and therapeutic approach. Gynecol Obstet (Sunnyvale). (2016) 6:376. 10.4172/2161-0932.1000376

[24] Onu A Aluh D Ikehi M. Prevalence and management of dysmenorrhea among secondary school adolescents in Enugu State, Nigeria. Res Square. (2020). 10.21203/rs.3.rs-109221/v1

[25] Ballantyne JC Cousins MJ. Primary dysmenorrhea: an urgent mandate. (2013). https://api.semanticscholar.org/CorpusID:269398699

[26] Chen CX Shieh C Draucker CB Carpenter JS. Reasons women do not seek health care for dysmenorrhea. J Clin Nurs. (2018) 27(1–2):e301–8. 10.1111/jocn.13946 28681499 PMC5746430

[27] Derseh B Hailegiorgies K Afessa N Temesgen M. Prevalence of dysmenorrhea and its effects on school performance: a cross-sectional study. J Womens Health Care. (2017) 6(2):6. 10.4172/2167-0420.1000361

[28] Azagew AW Kassie DG Walle TA. Prevalence of primary dysmenorrhea, its intensity, impact and associated factors among female students’ at Gondar town preparatory school, Northwest Ethiopia. BMC Women’s Health. (2020) 20(1):5. 10.1186/s12905-019-0873-4 31906945 PMC6945628

[29] Ethiopian Minister of Education and Minister of Health. Ethiopia-School-Health-Program-Framework. Ministry of Health (2017). p. 27–32.

[30] Ju H Jones M Mishra G. The prevalence and risk factors of dysmenorrhea. Epidemiol Rev. (2014) 36:104–13. 10.1093/epirev/mxt009 24284871

[31] Mohammed H Hassen N Musa A. Dysmenorrhea and associated factors among secondary school students in East Hararghe zone, Eastern Ethiopia. East Afr. J. Health Biomed Sci. (2019) 3(1):39–48.

[32] Sung YT Wu JS. The visual analogue scale for rating, ranking and paired-comparison (VAS-RRP): a new technique for psychological measurement. Behav Res Methods. (2018) 50(4):1694–715. 10.3758/s13428-018-1041-8 29667082 PMC6096654

[33] Karout S Soubra L Rahme D Karout L Khojah HMJ Itani R. Prevalence, risk factors, and management practices of primary dysmenorrhea among young females. BMC Women’s Health. (2021) 21(1):392. 10.1186/s12905-021-01532-w 34749716 PMC8576974

[34] Mesele TT Ayalew HG Syoum AT Antehneh TA. Impact of dysmenorrhea on academic performance among Haramaya university undergraduate regular students, eastern Ethiopia. Front Reprod Health. (2022) 4:939035. 10.3389/frph.2022.939035 36303653 PMC9580782

[35] Acheampong K Baffour-Awuah D Ganu D Appiah S Pan X Kaminga A Prevalence and predictors of dysmenorrhea, its effect, and coping mechanisms among adolescents in Shai Osudoku District, Ghana. Obstet Gynecol Int. (2019) 2019:1. 10.1155/2019/5834159 PMC6545782 31236112

[36] Vodouhe M Imorou R Atade R Salifou K Vignonzan U Hounkponou N Prevalence and factors associated with dysmenorrhea in Parakou, Benin. Open J Obstet Gynecol. (2020) 10:1000–10. 10.4236/ojog.2020.1080095

[37] Habibi N Huang MS Gan WY Zulida R Safavi SM. Prevalence of primary dysmenorrhea and factors associated with its intensity among undergraduate students: a cross-sectional study. Pain Manag Nurs. (2015) 16(6):855–61. 10.1016/j.pmn.2015.07.001 26328887

[38] Mollazadeh S Sadeghzadeh Oskouei B Kamalifard M Mirghafourvand M Aminisani N Jafari Shobeiri M. Association between sexual activity during menstruation and endometriosis: a case-control study. Int J Fertil Steril. (2019) 13(3):230–5. 10.22074/ijfs.2019.5601 31310078 PMC6642425

[39] Shiferaw MT Wubshet M Tegabu D. Menstrual problems and associated factors among students of Bahir Dar university, Amhara national regional state, Ethiopia: a cross-sectional survey. Pan Afr Med J. (2014) 17:246. 10.11604/pamj.2014.17.246.2230 25309646 PMC4189866

[40] Zeru AB Muluneh MA. Thyme tea and primary dysmenorrhea among young female students. Adolesc Health Med Ther. (2020) 11:147–55. 10.2147/AHMT.S280800 33117031 PMC7585774

[41] Zhang X Zhang R Chen D Huang R Tian Y Zhang P Association of tea drinking and dysmenorrhoea among reproductive-age women in Shanghai, China (2013–2015): a cross-sectional study. BMJ Open. (2019) 9(4):e026643. 10.1136/bmjopen-2018-026643 30962237 PMC6500245

